# The Strategies Microalgae Adopt to Counteract the Toxic Effect of Heavy Metals

**DOI:** 10.3390/microorganisms13050989

**Published:** 2025-04-25

**Authors:** Xin-Yue Yang, Yu-Xin Wei, Yan-Qiu Su, Zhong-Wei Zhang, Xiao-Yan Tang, Yang-Er Chen, Ming Yuan, Shu Yuan

**Affiliations:** 1 College of Resources, Sichuan Agricultural University, Chengdu 611130, China; yang16970319@163.com (X.-Y.Y.); weiyx12@126.com (Y.-X.W.); zzwzhang@126.com (Z.-W.Z.); xytang@sicau.edu.cn (X.-Y.T.); 2 College of Life Science, Sichuan Normal University, Chengdu 610066, China; snowdream215@163.com; 3 College of Life Science, Sichuan Agricultural University, Ya’an 625014, China; anty9826@163.com (Y.-E.C.); yuanming@sicau.edu.cn (M.Y.)

**Keywords:** biomass production, biosorption, coupled algal system, microalgae, heavy metal removal

## Abstract

Besides biomass production, some microalgae have been used to treat wastewater contamination. However, in general, high concentrations of heavy metals significantly inhibit algal growth. We thus need to find ways to promote the resistance of microalgae to heavy metals, increase their growth rate under stress, and achieve coupling of heavy metal removal and biomass production simultaneously. In this review, mechanisms for removal of heavy metals by microalgae are proposed. Effects of exogenous chemical additives (dissolved organic matters, formaldehyde, sulphate, phosphate, nitric oxide donors, etc.) on algal biosorption to heavy metals are summarized. Genetic manipulation and microalgal strain selection strategies are also introduced, especially for the acid-tolerant strains with high biosorption efficiencies to Cr(VI) and Cd^2+^ at low pH conditions. Recent advances in (semi)continuous heavy-metal-bioremediation and biomass-production coupled system with immobilized microalgae, as well as challenges and solutions to the commercialization and industrialization of the coupled system were discussed.

## 1. Introduction

Heavy metals (HMs) like chromium (Cr), cadmium (Cd), arsenic (As), copper (Cu), zinc (Zn), nickel (Ni), mercury (Hg) and lead (Pb), usually and persistently exist in environments and have toxicity to humans [1]. HMs threatens ecosystem stability seriously because of their highly toxic, non-degradable, and bio-accumulative features, which has received much attention [2]. HMs in the water often exist in forms of ionic or precipitated statuses. Heavy metal ions are especially toxic to organisms compared to the other forms because they are highly soluble and chemically reactive. Thus, the treatments of HM contaminants mostly include the transfer of HMs in various media and the conversion of HMs in different forms to decrease diffusivity or toxicity [3].

Microalgae are the primary producers in the water ecosystem and belong to several species groups comprising both photosynthetical prokaryotes and eukaryotes. Photosynthetical extremophiles are mainly green algae and red algae [4,5,6]. Microalgae offer a good biological generator for resource recycling and carbon fixation via nutrition assimilation, biomass harvesting, biofuel production, and further generation of other bioproducts [7,8,9,10].

Besides biomass production, some microalgae have been used to treat wastewater. The microalgae absorb nutrients, e.g., nitrogen (ammonium and nitrate) and phosphate, and reduce the contents of toxic chemicals, such as HMs and antibiotics [11,12]. As a defense strategy, microalgae synthesize higher levels of unsaturated fatty acids upon HM treatments [13]. For instance, Das et al. [14] recently indicated that the mixed microalgal consortium (mainly including *Chlorella vulgaris* and *Chlorella sorokiniana*) can be used for the removal of HMs in the photosynthetic microbial fuel cell (PMFC). An increase in algal lipid accumulation has been found during the HM removal. They showed that under 50 mg/L Cu or Co treatments, lipid yield was increased by 39.3% or 36.5%, respectively. However, too high concentrations of HMs inhibit algal growth significantly. For example, 2 mg/L Cu inhibited the growth of *Desmodesmus* sp. [indicated by chlorophyll (Chl) a content] by 50%, while 12 mg/L Cu caused *Desmodesmus* sp. to die [15]. So, we need to find ways to promote the resistance of microalgae to HMs, increase their growth rates under the stress, and achieve coupling of HM removal and biomass production simultaneously.

Although the mechanisms for removal of HMs by microalgae have been reviewed, biochemical or genetic engineering strategies to improve microalga’s tolerance to HMs have not been summarized comprehensively so far. Exogenous chemical additives, genetic manipulation, microalgal strain selection strategies, and immobilization methods are included in this review. Furthermore, recent advances in cost-effective (semi)continuous HM-bioremediation and biomass-production coupled system with immobilized microalgae were discussed.

## 2. Mechanisms for Removal of Heavy Metals by Microalgae

Microalgae can remove HMs through either biosorption or bio-accumulation. Biosorption is metabolism-independent but fast, while bioaccumulation is metabolism-dependent but slow [16]. Biosorption could be attributed to two mechanisms: adsorption onto the cell wall and the extracellular polymeric substances (EPS), both of which depend on the chemical interaction between the active groups and HMs [16,17,18,19]. The typical algal cell walls consist of a cellulose complex binding with mannans or xylans and a matrix that is structured with sulfated galactans, alginate, and alginic acids [16,17]. Similarly, EPS are also mainly composed of glycoproteins and polysaccharoses, and they also present many chemically active groups [18,19].

**Figure 1:**
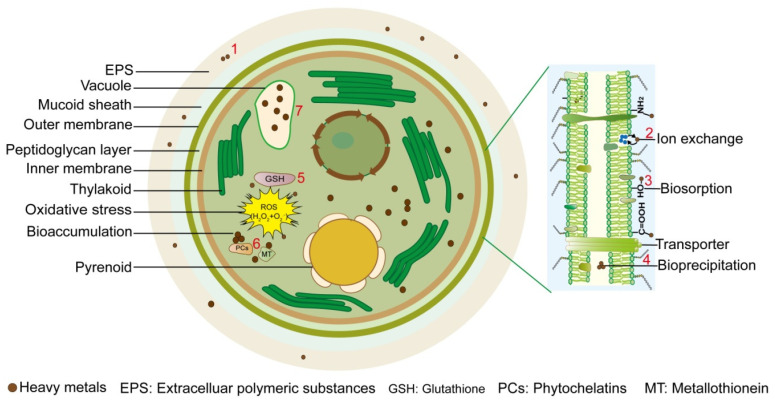
Biochemical mechanisms for removal of heavy metals by microalgae. Seven biosorption or bio-accumulation processes are summarized: (1) microalgal extracellular polymeric substances (EPS) contain polysaccharides, proteins, lipids, and alginates, which adsorb heavy metal (HM) ions through non-polar interactions, such as van der Waals forces and hydrogen bonds; (2) metal ions on the cell membrane, such as calcium, sodium, and potassium, can undergo ion exchange reactions with HM ions, allowing them to enter the cell; (3) the abundant negatively charged chemical groups such as -OH, -COOH, and -NH_2_, contained on the cell membrane and cell walls of microalgae attract positively charged HM ions through electrostatic attraction; (4) the adsorbed HM ions can undergo chemical reactions with negatively charged ions on the cell membrane, forming biological precipitates such as lead phosphate, cadmium phosphate, and cadmium sulfide, which may accumulate in the periplasmic space; (5) HM ions enter cells through active transport or passive diffusion on the cell membrane, producing a large amount of ROS. To cope with these oxidative damages, microalgae produce a large amount of glutathione (GSH) to eliminate ROS; (6) intracellular phytochelatin (PC) and metallothionein (MT) bind with HM ions to form protein-HM complexes, reducing their toxicity; (7) HM ions may be sequestered into the microalgal vacuoles, where organic acids, proteins, and other substances can also bind with HM ions to achieve detoxification.

In the biosorption process, the microalgal cell wall is the first barrier met by HMs. Proteins and polysaccharoses present in the microalgal cell wall contain a lot of HM binding sites [20]. Because of the variant contents and distribution of cell wall components in various microalga species, the biosorption capacities to different HMs vary largely. Alginate polymers are the main cell wall components that absorb HM ions in microalgae, and the adsorption capacities are mainly correlated with the number of binding sites on the polymers [21,22,23,24]. The chemically active groups of polysaccharoses and glycoproteins contribute to the bio-adsorption of microalgae [25]. Phosphate groups, amine groups, hydroxyl groups, and carboxyl groups are the major chemically active groups on the cell wall, which make the cell wall surface electro-negative [26,27]. These groups could bind with HMs via multiple ways such as electrostatic interaction, surface precipitation, complexation and ion exchange [28,29,30]. EPS also plays an important role in the biosorption process. For example, the adsorption of EPS on the MoS_2_ surface reduced the attachment sites of MoS_2_, making MoS_2_ less likely to be enriched inside the algal cell [31] (Figure 1).

The adsorbed heavy metal ions can undergo chemical reactions with negatively charged ions on the cell membrane, forming heavy metal biological precipitates such as lead phosphate, cadmium phosphate, cadmium sulfide, etc., which then accumulate inside the cytoplasm and the periplasmic space [32,33,34].

Bioaccumulation means the transport of HMs across cell walls and cytomembranes and the subsequent storage inside the cell [35]. This is a time-consuming process and usually happens in living cells, because that it is also an energy-consuming process [36,37,38]. The bio-accumulation typically occurs after the adsorption by EPS and the cell wall, with a part of adsorbed HMs transporting into the cell through some uptaking pathways named “membrane transport mechanism”. So far, the entry pathways of HMs into the cell could be separated into two routes: direct transport (ATP-relied) and indirect transport (by ligands). After reaching the cytosol, HMs interact with organic chemicals and metal-binding proteins before transporting into the vacuole [39,40]. Bio-accumulation would only occur in the living cell, and thus the biosorption effects are positively related with the cell viability.

Intracellular accumulation of HMs leads to reactive oxygen species (ROS) generation, which damages photosynthetic apparatus, and inhibits algal growth [41]. For example, of *Dunaliella salina*, HM stresses induced oxidative damages and more requirements for the synthesis of antioxidants and antioxidant enzymes [41]. Glutathione (GSH), a well-documented ROS-induced peptide that scavenges free radicals upon environmental stresses, is a sulfocompound made of 3 amino acids and provides major non-protein thiols in almost all organisms [42]. Residual cysteine in GSH contains sulfhydryl groups showing high affinity to HMs [43]. GSH is a precursor to the phytochelatin (PC) [44]. While PC is a kind of peptide composed of γ-Glu-Cys di-peptide repeats followed by the terminal Gly and induced by multiple HM stresses [45]. Induction of PC synthase in algae seems to be generally non-specific for HM types. However, some metal ions induce more PCs than others relying on the microalgal species [45]. The PCs could chelate HMs and therefore reduce their toxicity through reacting with cysteinyl thiols [46,47]. Then, the non-toxic HMs may be sequestered into the vacuole [48]. Nevertheless, the PC content was not always correlated with metal biosorption capacity, which implies that other mechanisms may also be involved in HM detoxification [49]. For example, metallothioneins have such a function [50,51,52] (Figure 1).

## 3. Microalgae Show High Removal Efficiencies to Low Levels of Heavy Metals

Zeraatkar et al. [53] collected the literature before 2014 about biosorption capacities of 14 different heavy metal ions by various microalga species in the optimal conditions. The maximum biosorption capacity ranged from 0.6 mg/g for Ni(II) to 836.5 mg/g for Zn. However, their removal rates were not always measured. Here, we summarized removal efficiencies to HMs by algae retrieved from the literatures after 2014 (Table 1).

The flocculating *Chlorella vulgaris* JSC-7 showed both higher biosorption capacity and higher removal rate to Zn and Cd than the non-flocculating *Chlorella vulgaris* CNW11, which may be because of its unique cell wall components [54]. *Chlorella*, *Scenedesmus*, and *Chlamydomonas* demonstrated high removal efficiencies and tolerances to many kinds of HMs. For 500 mg/L Tl^+^, *Chlorella vulgaris*, *Chlamydomonas reinhardtii*, and *Scenedesmus acuminatus* exhibited high removal efficiencies of 96%, 95%, and 87%, respectively [55]. *Desmodesmus* demonstrated high removal efficiencies for Cu and Ni mixtures, implying a synergistically removal capability for multiple HMs by the microalga [15]. In their report, the removal efficiencies for Cu were higher than those of Ni for all the mixed solutions with a maximum rate of 95%. *Scenedesmus almeriensis* was sensitive to Boron (B; a metalloid element). Cell density only reached 56% of the control at 60 mg/L of B and below 20% at higher levels of B. Contrastingly, *Chlorella vulgaris* was the rather tolerant to B with no growth arresting even at 120 mg/L B, though 180 mg/L B arrested *Chlorella vulgaris* growth completely [56]. *Chlorella vulgaris* showed 100%, 74%, 38%, and 26% removal efficiencies for 0.1, 0.3, 0.6, and 0.9 mg/L Cu, respectively [57]. *Scenedesmus acutus* and *Chlorella pyrenoidosa* also exhibited relatively high Cd^2+^ removal capabilities [58]. Furthermore, *Chlorella pyrenoidosa* can also remove Pb and Cu ions with removal rates of 72.86% for 3.64 mg/L Pb and 73.39% for 3.27 mg/L Cu, respectively [59]. The adsorption efficiencies of *Parachlorella kessleri* to Cerium (Ce), Gadolinium (Gd), and Lanthanum (La) exceeded 48% at concentrations of 100 μg/L to 1 mg/L [60]. In addition, these rare earth elements promoted the accumulation of mono-unsaturated fatty acids and saturated fatty acids in the alga [60]. Some *Botryocossuss* and *Chlorella* species showed high removal efficiencies for Cr(VI) [61,62,63], while *Scenedesmus aldavei*, *Desmodesmus pannonicus*, *Chlorella vulgaris*, *Chlorella sorokiniana*, and *Chlamydomonas reinhardtii* showed high removal efficiencies for Mo(VI) [27,31,64,65,66] (Table 1).

In summary, a clearance rate of over 80% is generally only applicable to heavy metal concentrations below 3 mg/L. Once the concentration of heavy metals exceeds 10 mg/L, it would inhibit microalgal growth severely, and the removal efficiencies usually drop below 50% under these conditions.

## 4. Exogenous Chemical Additives

Some chemical additives may enhance algal growth and/or biosorption capacity to HMs significantly. Both ethylene diamine tetraacetic acid (EDTA) and fulvic acid (FA) decreased the toxicity of Cu to *Scenedesmus subspicatus* through a mechanism of preventing cupric ions from being absorbed by the cell walls [67]. Dissolved organic matters (DOMs), like FA, citric acid, and humic acid (HA) reduced the bio-availability of Cu to *Chlorella pyrenoidosa* because of its complexation with Cu [67]. On the contrary, DOMs enhanced the biosorption of Pb to *Chlorella pyrenoidosa* via the formation of a ternary Pb-DOM complex on the algal surface, which resulted in higher accumulation of Pb [68]. For *Chlorella vulgaris*, FA treatments also increased the specific growth rate by 10% under 0.5 mg/L Cr, and increased the removal rate from 54% to 62% [69].

FA is featured by its high level of oxygen-containing active groups, e.g., carboxyl groups (-COOH), alcohol groups (-OH), and phenolic hydroxyl groups (-Ph-OH). On the other hand, it has a high solubility and a strong ROS-scavenging ability [70]. FA largely promoted the extracellular adsorption of Cr by *Chlorella vulgaris*, enhanced the removal of Cr by the alga, and therefore alleviated the arresting effects of Cr(VI) on *Chlorella vulgaris* growth [69]. Low-concentration FA may both be used as a carbon source to enhance the proliferation and growth of the alga, and provide a large number of adsorption sites through secreting EPS and increasing the number of chemically acidic groups (hydroxyl and carboxyl) (Table 2).

Organic acids enhance acidity of the medium, while pH has a key role in Cr(VI) biosorption. For example, at the optimal pH of 2.0, *Chlorella vulgaris* achieved the maximum adsorption [64]. Hexavalent Cr may form a surface complex with some protonated chemical group on the biosorbent, e.g., -NH_2_, -COOH, or -SO_3_H. The Cr(VI) anion may also be reduced to the Cr(III) cation by oxidizing the secondary alcohol group on the biosorbent. Then, the reduced Cr(III) may form an organic-metal complex through ion exchange or a coordination reaction. Under acidic conditions, hexavalent chromium is easily reduced to trivalent chromium, and the optimal pH is generally between 2 and 3 [64,71,72].

In cultures with cadmium, the sulphate additive promoted the *Chlamydomonas moewusii* growth [73]. The mechanism may be that the intracellular GSH has been consumed during the PC biosynthesis and replaced rapidly by de novo synthesis from the sulfur in the medium. The thiols allow PCs to bind with HMs and thereby to construct a PC-metal complex [74]. The incorporation of Cd into PC-Cd complexes leads to S_2_Cd crystal formation, which may store Cd more efficiently, and the thiol-containing peptides act as a coating on the crystal [73,75] (Table 2).

Our previous study also showed that 2.0 g/L metabisulfite increased the reduction rate of photosynthetic bacterium *Rhodobacter sphaeroides* SC01 from about 50% to 91% for 500 mg/L Cr^6+^ at 96 h [33]. Chemically active groups, like -OH, -PO_3_, -COOH, -CONH, -SO_3_, and -S-S- may play a key role in the Cr^6+^ adsorption. Then, Cr(III) reduced by the microorganism may be bio-precipitated in the formation of CrPS_4_ and Cr_2_P_3_S_9_ [33,34]. *Rhodobacter palustris* SC06, is a sulfate-oxidizing bacterium, who could transform SO_4_^2−^ to S^2−^, therefore precipitating with HMs [76,77]. *Desulfobacterota* can transform HMs into sulfide-metal precipitates, e.g., PbS and CdS [78,79]. Although *Rhodobacter* and *Desulfobacterota* are not eukaryotic microalgae, similar HM precipitates may also be formed on eukaryotic algal surface, which requires further investigations.

In addition, a study showed that phosphate also played a role in HM adsorption in a microalgal-bacterial symbiosis system. Zn^2+^ and phosphate may form a chemical precipitate, which decreased the fixation of above microbial system for Zn^2+^ via intracellular uptake and extracellular adsorption [80] (Table 2). Our previous study also indicated that bio-precipitation and reduction Cr(VI) in *Rhodobacter sphaeroides* were enhanced by the addition of (NaPO_3_)_6_ or Na_4_P_2_O_7_ salts. As a result, CrPO_4_∙6H_2_O or Cr_5_(P_3_O_10_)_3_ precipitate was identified by the X-ray diffraction analysis [32,81].

Salicylic acid (30–60 mg/L) induced 60–100% increases in biomass of *Scenedesmus obliquus* and *Chlorella pyrenoidosa* at 3.0 mg/L Cd for 96 h [82]. Treatments with homoserine lactones (analogs of bacterial quorum-sensing signaling molecules) enhanced Chl content by 10% at 100 μg/L Cd in a microalgal-bacterial consortia [83,84]. Although nitric oxide (NO) donor sodium nitroprusside (SNP) did not increase biomass, it enhanced algal lipid content from 51% to 60% at 5 μg/L Tl^+^ (the control microalgae without Tl^+^ or SNP contained only 38% lipids) [85]. Other algal growth regulators are subjected to further studies.

**Table 2:** Effects of exogenous additives on microalgal growth or HM biosorption capacity.

Microalga Species	Additives	Effects on Algal Growth or HM Biosorption Capacity	Reference
*Scenedesmus subspicatus*	EDTA Fulvic acid	Significantly reduce the concentration of Cu adsorbed by the cell wall	[67]
*Chlorella pyrenoidosa*	Citric acid	Removal rate increased from 81% to 87% for 0.0016–0.025 mM Cu	[68]
	Fulvic acid	Removal rate increased from 81% to 87% for 0.0016–0.025 mM Cu	
	Humic acid	Removal rate increased from 81% to 88% for 0.0016–0.025 mM Cu	
*Chlorella vulgaris*	Fulvic acid	Specific growth rate increased by 10% under 0.5 mg/L Cr; removal rate increased from 54% to 62%	[69]
*Chlamydomonas moewusii*	Sulfate ions	1 mM sulphate increased EC_50_ from 0.5 mg Cd/L to 4.46 mg Cd/L	[73]
*Multiple microalgae*	Phosphate	100% higher Chl content at 5 mg/L ZnSO_4_·7H_2_O in a microalgal-bacterial symbiosis system	[80]
*Chlorella pyrenoidosa*	Salicylic acid	60% higher cell density at 3.0 mg/L Cd and 96 h	[82]
*Chlorella vulgaris*	Homoserine lactones	10% higher Chl content at 100 μg/L Cd in a algae-bacteria consortia	[84]
*Parachlorella kessleri* R-3	Sodium nitro-prusside (SNP)	Lipid content increased from 51% to 60% at 5 μg/L Tl^+^ (control content was 38%)	[85]

## 5. Genetic Manipulation

Key genes related with HM uptake, metabolism, or detoxication in microalgae have been identified [66]. The most common genetic manipulation technique for algal-related metal removal is construction of transgenic algal strains by over-expressing endogenous or exogenous genes [86]. The microalgal cell is transformable and its genome may be reprogrammed to show a desired feature through using the suitable delivery system for transgenesis [87]. Genetic engineering has been proved to be a powerful tool to increase the capability of many microbes to remediate HMs [88].

For examples, *Chlamydomonas reinhardtii* expressing a foreign class-II metallothionein showed one-time higher cell density at 40 μM Cd stress [50,51]; *Chlamydomonas reinhardtii* expressing a mothbean Δ^1^-pyrroline-5-carboxylate synthetase (P5CS) showed 75% higher cell density at 100 μM Cd [89]; and *Chlamydomonas reinhardtii* over-expressing an endogenous metal tolerance protein CrMTP4 showed 50% higher cell density at 0.4 mM Cd [90] (Table 3).

Most microalgae, like *Chlorella* and *Chlamydomonas*, are sensitive to mercury and can hardly remove it in the wastewater. A dose of 5 μM HgCl_2_ almost completely inhibited the growth of the wild-type *Chlorella* sp. DT, while the transgenic group with expression of a *Bacillus megaterium* mercuric reductase (MerA) increased chlorophyll content by 3–4 times at 24 h. The transgenic modification enhanced the removal rate from <1% to 68% for 40 μM Hg [91]. Similarly, *Chlamydomonas reinhardtii* expressing a surface-displayed metalloregulatory protein MerR showed five folds higher Hg^2+^ accumulation at 10^−9^ to 10^−7^ M Hg^2+^ treatments [92] (Table 3).

So far, limited microalga species have been subjected to transgenesis. *Chlamydomonas reinhardtii* is the most commonly used microalga for genetic manipulation, because its genome has been sequenced and a proper gene delivery system has been developed (bioremediation and bioproduct production in *Chlamydomonas* have been reviewed comprehensively [66]). Except for *Chlamydomonas*, proper delivery systems to other microalga species need to be developed.

## 6. Microalgal Strain Selection

Torricelli et al. [42] screened Cd-tolerant *Scenedesmus acutus* strains and found that their growth inhibition rate decreased from 82% to 58% at 4.5 μM Cd, compared with the ancestral strain. Mechanism studies of the Cd-tolerant strain showed a higher content of cysteine and high levels of both reduced GSH and phytochelatin [42]. After microalgal strain selection, IC_50_ of the resistant strains of *Dyctiosphaerium chlorelloides* increased by 18 times for K_2_Cr_2_O_7_ and 208 times for K_2_CrO_4_ [93].

Samadani et al. [94] demonstrated that a low-pH-tolerant alga, *Chlamydomonas* CPCC 121, previously identified from the water near a copper mine, tolerated higher levels of cadmium at pH 4, compared with the non-acidophilic strain. The exclusion of Cd on *Chlamydomonas* CPCC 121 surface was much higher at pH 4 than at pH 7. Abinandan et al. [95,96,97] also observed that, when grown at 2 mg/L Cd, the low-pH-tolerant strains *Heterochlorella* sp. MAS3 and *Desmodesmus* sp. MAS1 accumulated more Cd from the solution at pH 3.5, although their sensitivities to Cd were different [97]. On the other hand, Cd stresses promoted lipid production. Lipid contents were doubled in both MAS1 and MAS3 strains when cultured at pH 3.0 [95] (Table 4).

A test of algal diversity in Uranium (U) mining sites in Spain identified a few microalga species that adapted and colonized rapidly in U-polluted water [6,98]. Baselga-Cervera et al. [99] found some U-tolerant *Chlamydomonas* species that inhabited high U tailings ponds at this mining site. An extreme-U-tolerant strain was selected. They found that the ancestral strain ChlSP exhibited a U-uptake ability of 4.30 mg/g, while the selected strain ChlSG exhibited a U-uptake ability of 6.34 mg/g [99]. Furthermore, the U-uptake kinetics confirmed that the ChlSG strain could remove up to 4 mg/L U in 24 days (Table 4).

Similarly, Beaulier et al. [100] isolated a U-tolerant *Coelastrella* strain from U-polluted water, and indicated that *Coelastrella* sp. PCV was much more resistant to uranium than *Chlorella vulgaris* or *Chlamydomonas reinhardtii*. The PCV strain had a capability to accumulate 25–55% of the U from the polluted water and then slowly release it to the medium, therefore limiting its toxic effects. Interestingly, some big lipid droplets were observed in cytoplasm of the PCV strain grown in U-polluted water [100], implying an enhanced lipid production, although the absolute lipid content was not quantified.

**Table 4:** Selected microalga strains exhibit better growth or higher HM biosorption capacities.

Microalga Species	Types of HMs	Effects on Algal Growth or HM Biosorption Capacity	Reference
*Scenedesmus acutus*	Cd	Inhibition rate of growth decreased from 82% to 58% at 4.5 μM Cd	[42]
*Dyctiosphaerium chlorelloides*	Cr	IC_50_ of K_2_Cr_2_O_7_ increased by 18 times; IC_50_ of K_2_CrO_4_ increased by 208 times	[93]
*Chlamydomonas* CPCC 121	Cd	10–25% higher relative cell division rate than the control strain at 100–600 μM Cd	[94]
*Desmodesmus* sp. MAS1	Cd	strain MAS1 was tolerant 20 mg L^−1^ Cd; Control strain MAS3 was tolerant 5 mg L−1 Cd	[97]
*Chlamydomonas reinhardtii*	U	Ancestral strain of 4.30 mg U g^−1^ DW; Selected strain of 6.34 mg U g^−1^ DW	[99]
*Coelastrella* sp. PCV	U	25–55% removal of 70–1100 ng in 20 mL culture medium	[100]

## 7. Immobilization Methods

Immobilized algae showed an increase in biosorption capacity relative to free algal cells by preventing loss of biomass during the biosorption cycle [53]. Techniques such as flocculation, adsorption on surfaces, covalent binding to carriers, crosslinking of algal cells, and entrapment of algae in polymeric matrix have been used for alga immobilization [53]. Zeraatkar et al. [53] collected the literatures before 2014 about biosorption capacities of immobilized algae to HMs. In general, immobilization increased the maximum sorption for 2.1–3.1 folds. Recently, some biopolymers used for algal immobilization have been developed. For example, 5 forms of ligno-cellulosic materials composed of pine sawdust, rick husk or sugarcane bagasse have been designed as the bio-carriers for low-cost microalgal biofilm culturing of 3 microalga species: *Diplosphaera* sp., *Hydrodictyon reticulatum*, and *Chlorella vulgaris* [101]. Pine sawdust was considered to be the best carrier for biomass accumulation and the immobilization with pine sawdust enhanced the content of saturated fatty acids from 48.71% to 55.58–57.08%. Interestingly, the pine sawdust leachate also increased *Chlorella vulgaris* growth, because the bio-carrier may also be leached into the culture medium, and the energy conversion propriety may be improved due to the declined crystallinity and the decreased ash content [101]. Unfortunately, removal capabilities of these immobilized algae to HMs were not tested in the report. *Chlorella vulgaris* immobilized with pine sawdust might be a good system for HM removal.

A new biofilm (AlgaPol) made of *Chlorella sorokiniana* and renewable copolymers produced by inverse vulcanization [102] was developed for its capability to removal HMs [103]. AlgaPol biofilm was able to remove 8 mg/L Cd^2+^ or Cu^2+^ from the growth medium with efficiencies of >90%.

Biochar has been widely used in removal of HM pollution with its advantages of high efficiency, low cost, and long-term binding [104,105,106]. However, in most cases, biochar and algae were used separately as the adsorbents to treat HM-polluted water. Jiang et al. [107] invented a biochar-alga complex of activated carbon derived from Coconut shells and *Chlorella*, and studied its adsorption capability to As and Hg ions. The new biochar-alga complex adsorbed up to 46.8 μg/g at the initial concentration of 0.1 mg/L Hg.

Electrochemical techniques have also been introduced in immobilized microalgae. A new dielectrophoresis-assisted device for the removal of HMs by culturing *Chlorella* alga was presented [108]. To generate the electric force, pairs of electrode mesh were included in the system, and an in-homogeneous electric field gradient was generated. The electrode mesh limited movement of the alga. Therefore, it can be considered as a semi-immobilized device. In a Cd^2+^ and Cu^2+^ mixture (0.5 mg/L for each), the individual adsorption efficiency of copper and cadmium achieved 98% and 96%, respectively. By regulating the electric voltage and the electrode size, the removal efficiency of *Chlorella* reached up to 97% [108].

The utilization of microalgae in photosynthetic microbial fuel cell (PMFC) is another strategy for HM removal. A modular PMFC was fabricated by Das et al. [14]. Mixed anaerobic sludge (algae were immobilized with volatile suspended solids) was adopted as inoculum for electrogenic microbiota and further cultured with synthetic wastewater with sucrose and HMs. Then, PMFC was operated in a fed-batch mode with 3–4 days of retention time in the cathodic chamber and anodic chamber, respectively. The removal efficiencies of Cu^2+^ and Co^2+^ achieved 94% and 88%, respectively, with an initial level of 50 mg/L. Interestingly, lipid production in the algal consortium increased by 1.2 and 1.1 times under Cu and Co stresses, respectively [14].

Other algal cultivation systems utilizing sludge have also been reported. The carriers enhance the substance exchange with HMs, and rise the surface to light and promote algal growth. In these systems, the suspended carriers, e.g., polyethylene carrier, were put into suspended biofilm reactors to form novel microalgal-bacterial symbiosis systems [3,109,110,111]. These microalgal-bacterial symbiosis systems showed high fixation capacities to HMs [80] (Table 2). Now bacterial and microalgae consortia are being increasingly used in bioremediation, such as the bacterial consortia in detoxification on *Chlamydomonas* [112]. Bacteria play an important role in promoting microalgal growth, enhancing bio-flocculation and facilitating cell wall disruption, and thus expanding the application potential of microalgal biofuel production. The major challenges to scale up microalgal-bacterial consortia and corresponding recommendations for further research have been addressed elsewhere [112].

Similarly, fungi–algae symbiotic systems have also been fabricated to immobilize algae and strengthen the bioadsorption of HMs. Wang et al. [113] reported a stable fungi-cyanobacteria symbiotic system and investigated its adsorption capability to Cd^2+^. The fixation efficiency of fungi to cyanobacteria reached up to 95% at the optimal condition. The Chl content, biomass, and fatty acids of the symbiotic system were significantly higher than those of fungi or cyanobacteria alone (lipid production increased by 1.2 times in the symbiotic system) [113].

## 8. Coupling HM Bioremediation and Biofuel Production

The potential of microalgae to remove HMs makes them a very promising tool for renewable and low-cost HM bioremediation [53,114,115]. Running and maintenance costs for algal lipid production could be greatly decreased by using HM-contaminated water [116]. Hybrid HM removal and algal culturing systems would decline the unit cost of energy by 20% to 25% and greatly reduce the consumption of freshwater and nutrient supplementations [117,118,119].

Microalgal cultivation in HM-contaminated water has been considered as an ecological restoration method for HM bioremediation, biomass production, and renewable energy generation [53,120]. Furthermore, a large kind of valuable by-products (e.g., biodiesel and bioethanol), nutrients, and value-added compounds could be isolated from the accumulated biomass [116]. Coupling HM bioremediation and lipid production could reduce the costs of microalgal biomass production, and remove toxic pollutants effectively, including HMs [114,121]. In addition, cultivation of microalgae would also decline the final cost of carbon dioxide sequestration from power plants or industrial sources [122]. Nevertheless, to obtain a high biomass and a high removal efficiency of a coupled algal system, the optimal autotrophic condition needs to be built. It would be feasible by using high rate microalgal ponds, which have been used globally in cost-effective HM treatments [117,123]. However, so far, large-scale coupled algal systems have been less reported. The basic challenge facing the commercialization and industrialization of the coupled system is the high costs for running and scale-up operation.

Most previous studies adopted discontinuous systems. Recently, a semi-continuous coupled algal system was conducted with a batch of immobilized algal beads (*Chlorella vulgaris* was immobilized in the alginate–calcium hydrogel) [124]. They described integrated processes with immobilized microalgae for HM bioremediation and algal biomass production, harvesting, and dewatering. High nutrient removal efficiencies were achieved both in continuous and semi-continuous systems. However, it was still laboratory-level research. The fabrication of continuous, high-efficiency, and large-scale algal operation systems require further investigations [29].

A major economic and technical problem associated with large-scale cultivation of microalgae, even in closed photobioreactors, is invasion by contaminating microorganisms. Avoiding this requires costly media sterilization or aseptic techniques [125]. Some non-axenic (non-sterile) cultivation systems of microalgae have been developed. However, most of them require high concentration of external carbon, nitrogen, or phosphorus supplies, which limited their applications [125,126,127,128]. So far, most HM-bioremediation and lipid-production coupled algal systems adopt sterile cultivation. The non-sterile and economically-feasible algal operation systems used for HM removal still need to be explored [129,130].

## 9. Conclusions

Coupling algal growth on bioremediation is a cost-effective strategy for achieving HM removal and biomass production simultaneously. However, high concentrations of HMs inhibit algal growth severely and microalgae show high removal efficiencies only to relatively low levels of HMs. Among exogenous chemical additives, sulphates showed promising effects of greatly promoting algal tolerance to HMs and NO donors enhanced lipid production under HM treatments. For Cr(VI) and Cd^2+^, low pH values greatly enhanced the biosorption. Thus, acid-tolerant strains could be selected for HM removal. For highly-toxic metal ions, like Hg^2+^, transgenic microalgae expressing functional genes may be adopted. Low-cost but high-efficient immobilization materials, as well as continuously large-scale coupled algal systems still need to be developed.

Until now, microalgae-based biofuel production coupled with HM removal is not economically feasible. However, major breakthroughs have been made in recent years towards design and development of advanced technologies able to increase product yields and at the same time to decrease processing costs. In order to achieve this goal, a variety of less investigated species of microalgae may be investigated, which can open the rooms for their adoption for biofuel production and HM removal by using modern biotechnology.

## Figures and Tables

**Table 1:** Removal efficiencies of microalgae for common HMs.

Microalga Species	Types of HMs	Removal Efficiency	Reference
*Chlorella vulgaris*	Cu	39% removal of 11.9 mg/L	[15]
*Desmodesmus* sp.	Cu	43% removal of 11.9 mg/L	
*Chlorella vulgaris*	Ni	32% removal of 5.7 mg/L	
*Desmodesmus* sp.	Ni	39% removal of 5.7 mg/L	
Flocculating *Chlorella vulgaris* JSC-7	Zn	89% for 20 mg/L	[54]
	Cd	62% for 4 mg/L	
Non-flocculating *Chlorella vulgaris* CNW11	Zn	40% for 20 mg/L	
	Cd	25% for 4 mg/L	
*Scenedesmus acuminutus*	Tl	100% for 150 mg/L; 91% for 250 mg/L; 87% for 500 mg/L	[55]
*Chlorella vulgaris*	Tl	100% for 150 mg/L; 89% for 250 mg/L; 96% for 500 mg/L	
*Chlamydomonas reinhardtii*	Tl	100% for 150 mg/L; 94% for 250 mg/L; 95% for 500 mg/L	
*Chlorella vulgaris*	Mn	99.4% for 3 mg/L	[56]
	Cu	87.9% for 3 mg/L	
	Zn	88.8% for 3 mg/L	
*Scenedesmus almeriensis*	As	40.7% for 12 mg/L	
	B	38.6% for 60 mg/L	
*Chlorella vulgaris*	Cu	100, 74, 38 and 26% for 0.1, 0.3, 0.6 and 0.9 mg/L	[57]
*Chlorella pyrenoidosa*	Cd	45.45% removal of 1.5 ppm	[58]
*Scenedesmus acutus*	Cd	57.14% removal of 1.5 ppm	
*Chlorella pyrenoidosa*	Pb	72.86% for 3.64 mg/L	[59]
	Cu	73.39% for 3.27 mg/L	
	Cd	48.42% for about 3 mg/L	
*Parachlorella kessleri* R-3	Ce	66.2% of 100 μg/L Ce^3+^	[60]
	Gd	48.4% of 250 μg/L Gd^3+^	
	La	59.9% of 1 mg/L La^3+^	
*Botryocossuss* sp. NJD-1	Cr(VI)	94.2% of 5 mg/L Cr(VI)	[61]
*Chlorella vulgaris* ZBS1	Cr(VI)	75.46% of 2.1 mg/L Cr(VI)	[62]
*Chlorella vulgaris*	Cr(VI)	60.38% of 5 mg/L Cr(VI) at pH 2	[63]
*Chlorella vulgaris*	Mo(VI)	80.3% of 0.5 mg/L Mo(VI)	[64]
*Chlorella sorokiniana* TU5	Mo(VI)	57.8% with 115.65 mg/L Mo(VI)	[65]

**Table 3:** Effects of genetic modification on microalgal growth or HM biosorption capacity.

Microalga Species	Genetic Modification Methods	Types of HMs	Effects on Algal Growth or HM Biosorption	Reference
*Chlamydomonas reinhardtii*	Expression of a class-II metallothionein	Cd	One-time higher cell density at 40 μM Cd	[50]
*Chlamydomonas reinhardtii*	Expression of metallothionein (MT)-like gene from *Festuca rubra*	Cd	IC_50_ of Cd increased by 55.43%	[51]
*Chlamydomonas reinhardtii*	Expression of a mothbean Δ^1^-pyrroline-5-carboxylate synthetase (P5CS)	Cd	Up to 75% higher cell density at 100 μM Cd	[89]
*Chlamydomonas reinhardtii*	Over-expression of metal tolerance protein CrMTP4	Cd	Cell density increased by 50% at 0.4 mM Cd	[90]
*Chlorella* sp. DT	Expression of a *Bacillus megaterium* strain MB1 mercuric reductase (MerA)	Hg	Removal rate increased from <1% to 68% for 40 μM Hg	[91]
*Chlamydomonas reinhardtii*	Expression of a surface displayed metalloregulatory protein MerR	Hg	Five folds higher Hg^2+^ accumulation at 10^−9^ to 10^−7^ M Hg^2+^	[92]

## Data Availability

No new data were created or analyzed in this study.

## References

[1] Singh D.V., Singh R.P. (2022). Algal consortia based metal detoxification of municipal wastewater: Implication on photosynthetic performance, lipid production, and defense responses. Sci. Total Environ..

[2] Zuo W.L., Yu Y.D., Huang H. (2021). Making waves: Microbe-photocatalyst hybrids may provide new opportunities for treating heavy metal polluted wastewater. Water Res..

[3] Zhou X.R., Wang R., Tang C.C., Varrone C., He Z.W., Li Z.H., Wang X.C. (2023). Advances, challenges, and prospects in microalgal-bacterial symbiosis system treating heavy metal wastewater. Chemosphere.

[4] Rothschild L.J., Mancinelli R.L. (2001). Life in extreme environments. Nature.

[5] Varshney P., Mikulic P., Vonshak A., Beardall J., Wangikar P.P. (2015). Extremophilic micro-algae and their potential contribution in biotechnology. Bioresour. Technol..

[6] Baselga-Cervera B., Garcia-Balboa C., Diaz-Alejo H.M., Costas E., Lopez-Rodas V. (2020). Rapid colonization of uranium mining-impacted waters, the biodiversity of successful lineages of phytoplankton extremophiles. Microb. Ecol..

[7] Watson J., Swoboda M., Aierzhati A., Wang T., Si B., Zhang Y. (2021). Biocrude oil from algal bloom microalgae: A novel integration of biological and thermochemical techniques. Environ. Sci. Technol..

[8] Chiu S., Kao C., Chen T., Chang Y., Kuo C., Lin C. (2015). Cultivation of microalgal *Chlorella* for biomass and lipid production using wastewater as nutrient resource. Bioresour. Technol..

[9] Mohsenpour S.F., Hennige S., Willoughby N., Adeloye A., Gutierrez T. (2021). Integrating micro-algae into wastewater treatment: A review. Sci. Total Environ..

[10] Kundu D., Dutta D., Samanta P., Dey S., Sherpa K.C., Kumar S., Dubey B.K. (2022). Valorization of wastewater: A paradigm shift towards circular bioeconomy and sustainability. Sci. Total Environ..

[11] Gojkovic Z., Lindberg R.H., Tysklind M., Funk C. (2019). Northern green algae have the capacity to remove active pharmaceutical ingredients. Ecotoxicol. Environ. Saf..

[12] León-Vaz A., León R., Giráldez I., Vega J.M., Vigara J. (2021). Impact of heavy metals in the microalga *Chlorella sorokiniana* and assessment of its potential use in cadmium bioremediation. Aquat. Toxicol..

[13] Nanda M., Jaiswal K.K., Kumar V., Verma M., Vlaskin M.S., Gururani P., Kim H., Alajmi M.F., Hussain A. (2021). Bio-remediation capacity for Cd(II) and Pb(II) from the aqueous medium by two novel strains of microalgae and their effect on lipidomics and metabolomics. J. Water Proc. Eng..

[14] Das S., Kumar S., Kumar Mehta A., Ghangrekar M.M. (2024). Heavy metals removal by algae and usage of activated metal-enriched biomass as cathode catalyst for improving performance of photosynthetic microbial fuel cell. Bioresour. Technol..

[15] Rugnini L., Costa G., Congestri R., Bruno L. (2017). Testing of two different strains of green microalgae for Cu and Ni removal from aqueous media. Sci. Total Environ..

[16] Naveed S., Li C., Lu X., Chen S., Yin B., Zhang C., Ge Y. (2019). Microalgal extracellular polymeric substances and their interactions with metal(loid)s: A review. Crit. Rev. Environ. Sci. Tec..

[17] Tripathi S., Poluri K.M. (2021). Heavy metal detoxification mechanisms by microalgae: Insights from transcriptomics analysis. Environ. Pollut..

[18] Pagliaccia B., Carretti E., Severi M., Berti D., Lubello C., Lotti T. (2022). Heavy metal biosorption by Extracellular Polymeric Substances (EPS) recovered from anammox granular sludge. J. Hazard. Mater..

[19] Li W.W., Yu H.Q. (2014). Insight into the roles of microbial extracellular polymer substances in metal biosorption. Bioresour. Technol..

[20] Schiewer S., Wong M.H. (2000). Ionic strength effects in biosorption of metals by marine algae. Chemosphere.

[21] Ubando A.T., Africa A.D.M., Maniquiz-Redillas M.C., Culaba A.B., Chen W.H., Chang J.S. (2021). Microalgal biosorption of heavy metals: A comprehensive bibliometric review. J. Hazard. Mater..

[22] Danouche M., El Ghachtouli N., El Arroussi H. (2021). Phycoremediation mechanisms of heavy metals using living green microalgae: Physicochemical and molecular approaches for enhancing selectivity and removal capacity. Heliyon.

[23] Spain O., Plöhn M., Funk C. (2021). The cell wall of green microalgae and its role in heavy metal removal. Physiol. Plant..

[24] Zhang H., Hu X., Li T., Zhang Y., Xu H., Sun Y., Gu X., Gu C., Luo J., Gao B. (2022). MIL series of metal organic frameworks (MOFs) as novel adsorbents for heavy metals in water: A review. J. Hazard. Mater..

[25] Yang T., Chen M.L., Wang J.H. (2015). Genetic and chemical modification of cells for selective separation and analysis of heavy metals of biological or environmental significance. Trends Anal. Chem..

[26] Priya A.K., Jalil A.A., Vadivel S., Dutta K., Rajendran S., Fujii M., Soto-Moscoso M. (2022). Heavy metal remediation from wastewater using microalgae: Recent advances and future trends. Chemosphere.

[27] Caner C., Erdaği D., Şeker B., Altundağ H., Çeti N.G., Tunca H. (2024). Effects of molybdenum to growth parameters and lipid content of two algae in *Scenedesmaceae taxa*. Heliyon.

[28] Li H.G., Watson J., Zhang Y.H., Lu H.F., Liu Z.D. (2020). Environment-enhancing process for algal wastewater treatment, heavy metal control and hydrothermal biofuel production: A critical review. Bioresour. Technol..

[29] Mustafa S., Bhatti H.N., Maqbool M., Iqbal M. (2021). Microalgae biosorption, bioaccumulation and biodegradation efficiency for the remediation of wastewater and carbon dioxide mitigation: Prospects, challenges and opportunities. J. Water Process Eng..

[30] Gondi R., Kavitha S., Yukesh Kannah R., Parthiba Karthikeyan O., Kumar G., Kumar Tyagi V., Rajesh Banu J. (2022). Algal-based system for removal of emerging pollutants from wastewater: A review. Bioresour. Technol..

[31] Cao M., Yang D., Wang F., Zhou B., Chen H., Yuan R., Sun K. (2023). Extracellular polymeric substances altered the physicochemical properties of molybdenum disulfide nanomaterials to mitigate its toxicity to *Chlorella vulgaris*. NanoImpact.

[32] Su Y.Q., Yuan S., Guo Y.C., Tan Y.Y., Mao H.T., Cao Y., Chen Y.E. (2021). Highly efficient and sustainable removal of Cr (VI) in aqueous solutions by photosynthetic bacteria supplemented with phosphor salts. Chemosphere.

[33] Su Y.Q., Min S.N., Jian X.Y., Guo Y.C., He S.H., Huang C.Y., Zhang Z., Yuan S., Chen Y.E. (2023). Bioreduction mechanisms of high-concentration hexavalent chromium using sulfur salts by photosynthetic bacteria. Chemosphere.

[34] Qian Z., Yanqiu S., Lin G., Hongmei D., Lihan Z., Shuangnan M., Shu Y., Yanger C., Qi L. (2024). Sulfur source promotes the biosorption and bioprecipitation of Cd in purple non-sulfur bacteria. Int. Biodeter. Biodegr..

[35] Leong Y.K., Chang J.S. (2020). Bioremediation of heavy metals using microalgae: Recent advances and mechanisms. Bioresour. Technol..

[36] Wang L., Liu X., Lee D.J., Tay J.H., Zhang Y., Wan C.L., Chen X.F. (2018). Recent advances on biosorption by aerobic granular sludge. J. Hazard. Mater..

[37] Beni A.A., Esmaeili A. (2020). Biosorption, an efficient method for removing heavy metals from industrial effluents: A review. Environ. Technol. Innov..

[38] Singh D.V., Bhat R.A., Upadhyay A.K., Singh R., Singh D.P. (2021). Microalgae in aquatic environs: A sustainable approach for remediation of heavy metals and emerging contaminants. Environ. Technol. Innov..

[39] Ghomi A.G., Asasian-Kolur N., Sharifian S., Golnaraghi A. (2020). Biosorpion for sustainable recovery of precious metals from wastewater. J. Environ. Chem. Eng..

[40] Kant Bhatia S., Ahuja V., Chandel N., Mehariya S., Kumar P., Vinayak V., Saratale G.D., Raj T., Kim S.H., Yang Y.H. (2022). An overview on microalgal-bacterial granular consortia for resource recovery and wastewater treatment. Bioresour. Technol..

[41] Bhuvaneshwari M., Thiagarajan V., Nemade P., Chandrasekaran N., Mukherjee A. (2018). Toxicity and trophic transfer of P25 TiO_2_ NPs from *Dunaliella salina* to *Artemia salina*: Effect of dietary and waterborne exposure. Environ. Res..

[42] Torricelli E., Gorbi G., Pawlik-Skowronska B., Di Toppi L.S., Corradi M.G. (2004). Cadmium tolerance, cysteine and thiol peptide levels in wild type and chromium-tolerant strains of *Scenedesmus acutus* (Chlorophyceae). Aquat. Toxicol..

[43] Ulrich K., Jakob U. (2019). The role of thiols in antioxidant systems. Free Radic. Biol. Med..

[44] Wang Y., Zhang C., Zheng Y., Ge Y. (2017). Phytochelatin synthesis in *Dunaliella salina* induced by arsenite and arsenate under various phosphate regimes. Ecotoxicol. Environ. Saf..

[45] Cobbett C.S. (2000). Phytochelatin biosynthesis and function in heavy-metal detoxification. Curr. Opin. Plant Biol..

[46] Noctor G., Queval G., Mhamdi A., Chaouch S., Foyer C.H. (2011). Glutathione. Arab. Book.

[47] Li M., Barbaro E., Bellini E., Saba A., Sanità di Toppi L., Varotto C. (2020). Ancestral function of the phytochelatin synthase C-terminal domain in inhibition of heavy metal-mediated enzyme overactivation. J. Exp. Bot..

[48] Tsuji N., Hirayanagi N., Okada M., Miyasaka H., Hirata K., Zenk M.H., Miyamoto K. (2002). Enhancement of tolerance to heavy metals and oxidative stress in *Dunaliella tertiolecta* by Zn-induced phytochelatin synthesis. Biochem. Biophys. Res. Commun..

[49] Clemens S. (2006). Evolution and function of phytochelatin synthases. J. Plant Physiol..

[50] Cai X.H., Brown C., Adhiya J., Traina S.J., Sayre R.T. (1999). Growth and heavy metal binding properties of transgenic *Chlamydomonas* expressing a foreign metallothionein gene. Int. J. Phytoremediat..

[51] Han S., Hu Z., Lei A. (2008). Expression and function analysis of the metallothionein-like (MT-like) gene from *Festuca rubra* in *Chlamydomonas reinhardtii* chloroplast. Sci. China C Life Sci..

[52] Balzano S., Sardo A., Blasio M., Chahine T.B., Dell’Anno F., Sansone C., Brunet C. (2020). Microalgal metallothioneins and phytochelatins and their potential use in bioremediation. Front. Microbiol..

[53] Zeraatkar A.K., Ahmadzadeh H., Talebi A.F., Moheimani N.R., McHenry M.P. (2016). Potential use of algae for heavy metal bioremediation, a critical review. J. Environ. Manag..

[54] Alam M.A., Wan C., Zhao X.Q., Chen L.J., Chang J.S., Bai F.W. (2015). Enhanced removal of Zn^2+^ or Cd^2+^ by the flocculating *Chlorella vulgaris* JSC-7. J. Hazard. Mater..

[55] Birungi Z.S., Chirwa E.M.N. (2015). The adsorption potential and recovery of thallium using green microalgae from eutrophic water sources. J. Hazard. Mater..

[56] Saavedra R., Muñoz R., Taboada M.E., Vega M., Bolado S. (2018). Comparative uptake study of arsenic, boron, copper, manganese and zinc from water by different green microalgae. Bioresour. Technol..

[57] Andrade L.M., Tito C.A., Mascarenhas C., Lima F.A., Dias M., Andrade C.J., Mendes M.A., Nascimento C.A.O. (2021). *Chlorella vulgaris* phycoremediation at low Cu^2+^ contents: Proteomic profiling of microalgal metabolism related to fatty acids and CO_2_ fixation. Chemosphere.

[58] Chandrashekharaiah P.S., Sanyal D., Dasgupta S., Banik A. (2021). Cadmium biosorption and biomassproduction by two freshwater microalgae *Scenedesmus acutus* and *Chlorella pyrenoidosa*: An integrated approach. Chemosphere.

[59] Rahmani A., Zerrouki D., Tabchouche A., Djafer L. (2022). Oilfield-produced water as a medium for the growth of *Chlorella pyrenoidosa* outdoor in an arid region. Environ. Sci. Pollut. Res. Int..

[60] Song X., Liu B.F., Kong F., Song Q., Ren N.Q., Ren H.Y. (2024). New insights into rare earth element-induced microalgae lipid accumulation: Implication for biodiesel production and adsorption mechanism. Water Res..

[61] Shen L., Saky S.A., Yang Z., Ho S.H., Chen C., Qin L., Zhang G., Wang Y., Lu Y. (2019). The critical utilization of active heterotrophic microalgae for bioremoval of Cr(VI) in organics co-contaminated wastewater. Chemosphere.

[62] Tattibayeva Z., Tazhibayeva S., Kujawski W., Zayadan B., Musabekov K. (2022). Peculiarities of adsorption of Cr (VI) ions on the surface of *Chlorella vulgaris* ZBS1 algae cells. Heliyon.

[63] Aththanayake A.M.K.C.B., Rathnayake I.V.N., Deeyamulla M.P., Megharaj M. (2022). Potential use of *Chlorella vulgaris* KCBAL01 from a freshwater stream receiving treated textile effluent in hexavalent chromium [Cr(VI)] removal in extremely acidic conditions. J. Environ. Sci. Health A Toxic Hazard. Subst. Environ. Eng..

[64] Urrutia C., Yañez-Mansilla E., Jeison D. (2019). Bioremoval of heavy metals from metal mine tailings water using microalgae biomass. Algal Res..

[65] Tambat V.S., Tseng Y.S., Kumar P., Chen C.W., Singhania R.R., Chang J.S., Dong C.D., Patel A.K. (2023). Effective and sustainable bioremediation of molybdenum pollutants from wastewaters by potential microalgae. Environ. Technol. Innov..

[66] Tejada-Jimenez M., Leon-Miranda E., Llamas A. (2023). *Chlamydomonas reinhardtii*—A reference microorganism for eukaryotic molybdenum metabolism. Microorganisms.

[67] Ma M., Zhu W., Wang Z., Witkamp G.J. (2003). Accumulation, assimilation and growth inhibition of copper on freshwater alga (*Scenedesmus subspicatus* 86.81 SAG) in the presence of EDTA and fulvic acid. Aquat. Toxicol..

[68] Shi W., Jin Z., Hu S., Fang X., Li F. (2017). Dissolved organic matter affects the bioaccumulation of copper and lead in *Chlorella pyrenoidosa*: A case of long-term exposure. Chemosphere.

[69] Luo L., Yang C., Jiang X., Guo W., Ngo H.H., Wang X.C. (2023). Impacts of fulvic acid and Cr (VI) on metabolism and chromium removal pathways of green microalgae. J. Hazard. Mater..

[70] Wu Y.J., Chen B.L. (2019). Effect of fulvic acid coating on biochar surface structure and sorption properties towards 4-chlorophenol. Sci. Total Environ..

[71] Kong D., Ma H., Zhu C., Hao Y., Li C. (2024). Unraveling the toxicity response and metabolic compensation mechanism of tannic acid-Cr(III) complex on alga *Raphidocelis subcapitata*. Sci. Total Environ..

[72] Luo L., Yang T., Dzakpasu M., Jiang X., Guo W., Ngo H.H., Wang X.C. (2024). Interplay of humic acid and Cr(VI) on green microalgae: Metabolic responses and chromium enrichment. J. Hazard. Mater..

[73] Mera R., Torres E., Abalde J. (2014). Sulphate, more than a nutrient, protects the microalga *Chlamydomonas moewusii* from cadmium toxicity. Aquat. Toxicol..

[74] Mehra R.K., Mulchandani P., Hunter T.C. (1994). Role of CdS quantum crystallites in cadmium resistance in *Candida Glabrata*. Biochem. Biophys. Res. Commun..

[75] Dameron C.T., Winge D.R. (1990). Characterization of peptide-coated cadmium-sulfide crystallites. Inorg. Chem..

[76] Li X.M., Peng W.H., Jia Y.Y., Lu L., Fan W.H. (2016). Bioremediation of lead contaminated soil with *Rhodobacter sphaeroides*. Chemosphere.

[77] Su Y., Shi Q., Li Z., Deng H., Zhou Q., Li L., Zhao L., Yuan S., Liu Q., Chen Y. (2024). *Rhodopseudomonas palustris* shapes bacterial community, reduces Cd bioavailability in Cd contaminated flooding paddy soil, and improves rice performance. Sci. Total Environ..

[78] Shan S., Guo Z., Lei P., Wang Y., Li Y., Cheng W., Zhang M., Wu S., Yi H. (2019). Simultaneous mitigation of tissue cadmium and lead accumulation in rice via sulfate- reducing bacterium. Ecotox. Environ. Saf..

[79] Peng W., Li X., Lin M., Gui H., Xiang H., Zhao Q., Fan W. (2020). Biosafety of cadmium contaminated sediments after treated by indigenous sulfate reducing bacteria: Based on biotic experiments and DGT technique. J. Hazard. Mater..

[80] Tang C.C., Hu Y.R., Zhang M., Chen S.L., He Z.W., Li Z.H., Tian Y., Wang X.C. (2024). Role of phosphate in microalgal-bacterial symbiosis system treating wastewater containing heavy metals. Environ. Pollut..

[81] Mao H.T., Chen L.X., Zhang M.Y., Shi Q.Y., Xu H., Zhang D.Y., Zhang Z.W., Yuan M., Yuan S., Su Y.Q. (2023). Melatonin improves the removal and the reduction of Cr(VI) and alleviates the chromium toxicity by antioxidative machinery in *Rhodobacter sphaeroides*. Environ. Pollut..

[82] Zhang T., Shi M., Yan H., Li C. (2022). Effects of salicylic acid on heavy metal resistance in eukaryotic algae and its mechanisms. Int. J. Environ. Res. Public Health.

[83] Li D., Liu R., Cui X., He M., Zheng S., Du W., Gao M., Wang C. (2021). Co-culture of bacteria and microalgae for treatment of high concentration biogas slurry. J. Water Process Eng..

[84] Yu Q., Chen J., Ye M., Wei Y., Zhang C., Ge Y. (2024). N-acyl homoserine lactones (AHLs) enhanced removal of cadmium and other pollutants by algae-bacteria consortia. J. Environ. Manag..

[85] Song X., Kong F., Liu B.F., Song Q., Ren N.Q., Ren H.Y. (2023). Thallium-mediated NO signaling induced lipid accumulation in microalgae and its role in heavy metal bioremediation. Water Res..

[86] Cheng S.Y., Show P., Lau F., Chang J., Ling T.C. (2019). New prospects for modified algae in heavy metal adsorption. Trends Biotechnol..

[87] Gimpel J.A., Henríquez V., Mayfield S.P. (2015). In metabolic engineering of eukaryotic microalgae: Potential and challenges come with great diversity. Front. Microbiol..

[88] Hassanien A., Saadaoui I., Schipper K., Al-Marri S., Dalgamouni T., Aouida M., Saeed S., Al-Jabri H.M. (2023). Genetic engineering to enhance microalgal-based produced water treatment with emphasis on CRISPR/Cas9: A review. Front. Bioeng. Biotechnol..

[89] Siripornadulsil S., Traina S., Verma D.P., Sayre R.T. (2002). Molecular mechanisms of proline-mediated tolerance to toxic heavy metals in transgenic microalgae. Plant Cell.

[90] Ibuot A., Dean A.P., McIntosh O.A., Pittman J.K. (2017). Metal bioremediation by CrMTP4 over-expressing *Chlamydomonas reinhardtii* in comparison to natural wastewater-tolerant microalgae strains. Algal Res..

[91] Huang C.C., Chen M.W., Hsieh J.L., Lin W.H., Chen P.C., Chien L.F. (2006). Expression of mercuric reductase from *Bacillus megaterium* MB1 in eukaryotic microalga *Chlorella* sp. DT: An approach for mercury phytoremediation. Appl. Microbiol. Biotechnol..

[92] Chokshi K., Kavanagh K., Khan I., Slaveykova V.I., Sieber S. (2024). Surface displayed MerR increases mercury accumulation by green microalga *Chlamydomonas reinhardtii*. Environ. Int..

[93] D’ors A., Pereira M., Bartolomé M.C., López-Rodas V., Costas E., Sánchez-Fortún S. (2010). Toxic effects and specific chromium acquired resistance in selected strains of *Dyctiosphaerium chlorelloides*. Chemosphere.

[94] Samadani M., Perreault F., Oukarroum A., Dewez D. (2018). Effect of cadmium accumulation on green algae *Chlamydomonas reinhardtii* and acid-tolerant *Chlamydomonas* CPCC 121. Chemosphere.

[95] Abinandan S., Subashchandrabose S.R., Cole N., Dharmarajan R., Venkateswarlu K., Megharaj M. (2019). Sustainable production of biomass and biodiesel by acclimation of non-acidophilic microalgae to acidic conditions. Bioresour. Technol..

[96] Abinandan S., Subashchandrabose S.R., Panneerselvan L., Venkateswarlu K., Megharaj M. (2019). Potential of acid-tolerant microalgae, *Desmodesmus* sp. MAS1 and *Heterochlorella* sp. MAS3, in heavy metal removal and biodiesel production at acidic pH. Bioresour. Technol..

[97] Abinandan S., Subashchandrabose S.R., Venkateswarlu K., Perera I.A., Megharaj M. (2019). Acid-tolerant microalgae can withstand higher concentrations of invasive cadmium and produce sustainable biomass and biodiesel at pH 3.5. Bioresour. Technol..

[98] García-Balboa C., Baselga-Cervera B., García-Sanchez A., Igual J.M., Lopez-Rodas V., Costas E. (2013). Rapid adaptation of microalgae to bodies of water with extreme pollution from uranium mining: An explanation of how mesophilic organisms can rapidly colonise extremely toxic environments. Aquat. Toxicol..

[99] Baselga-Cervera B., Romero-Lopez J., Garcia-Balboa C., Costas E., Lopez-Rodas V. (2018). Improvement of the uranium sequestration ability of a *Chlamydomonas* sp. (ChlSP Strain) isolated from extreme uranium mine tailings through selection for potential bioremediation application. Front. Microbiol..

[100] Beaulier C., Dannay M., Devime F., Galeone A., Baggio C., El Sakkout N., Raillon C., Courson O., Bourguignon J., Alban C. (2024). Characterization of a uranium-tolerant green microalga of the genus *Coelastrella* with high potential for the remediation of metal-polluted waters. Sci. Total Environ..

[101] Zhang Q., Yu Z., Jin S., Zhu L., Liu C., Zheng H., Zhou T., Liu Y., Ruan R. (2019). Lignocellulosic residue as bio-carrier for algal biofilm growth: Effects of carrier physicochemical proprieties and toxicity on algal biomass production and composition. Bioresour. Technol..

[102] Chung W.J., Griebel J.J., Kim E.T., Yoon H., Simmonds A.G., Ji H.J., Dirlam P.T., Glass R.S., Wie J.J., Nguyen N.A. (2013). The use of elemental sulfur as an alternative feedstock for polymeric materials. Nat. Chem..

[103] León-Vaz A., Cubero-Cardoso J., Trujillo-Reyes Á., Fermoso F.G., León R., Funk C., Vigara J., Urbano J. (2023). Enhanced wastewater bioremediation by a sulfur-based copolymer asscaffold for microalgae immobilization (AlgaPol). Chemosphere.

[104] Tao S., Li C., Fan X., Zeng G., Lu P., Zhang X., Wen Q., Zhao W., Luo D., Fan C. (2012). Activated coke impregnated with cerium chloride used for elemental mercury removal from simulated flue gas. Chem. Eng. J..

[105] Mohan D., Sarswat A., Ok Y.S., Pittman C.U. (2014). Organic and inorganic contaminants removal from water with biochar, a renewable, low cost and sustainable adsorbent—A critical review. Bioresour. Technol..

[106] Tao Q., Li B., Chen Y., Zhao J., Li Q., Chen Y., Peng Q., Yuan S., Li H., Huang R. (2021). An integrated method to produce fermented liquid feed and biologically modified biochar as cadmium adsorbents using corn stalks. Waste Manag..

[107] Jiang X., Zhang S., Yin X., Tian Y., Liu Y., Deng Z., Wang L. (2023). Contrasting effects of a novel biochar-microalgae complex on arsenic and mercury removal. Ecotox. Environ. Saf..

[108] Zhao K., Zhao X., Gao T., Li X., Wang G., Pan X., Wang J. (2023). Dielectrophoresis-assisted removal of Cd and Cu heavy metal ions by using *Chlorella* microalgae. Environ. Pollut..

[109] Tang C.C., Tian Y., Liang H., Zuo W., Wang Z.W., Zhang J., He Z.W. (2018). Enhanced nitrogen and phosphorus removal from domestic wastewater via algae-assisted sequencing batch biofilm reactor. Bioresour. Technol..

[110] Tang C.C., Tian Y., He Z.W., Zuo W., Zhang J. (2018). Performance and mechanism of a novel algal-bacterial symbiosis system based on sequencing batch suspended biofilm reactor treating domestic wastewater. Bioresour. Technol..

[111] Tang C.C., Wang T.Y., Zhang X.Y., Wang R., He Z.W., Li Z., Wang X.C. (2022). Role of types and dosages of cations with low valance states on microalgal-bacterial symbiosis system treating wastewater. Bioresour. Technol..

[112] Zhang B., Li W., Guo Y., Zhang Z., Shi W., Cui F., Lens P.N.L., Tay J.H. (2020). Microalgal-bacterial consortia: From interspecies interactions to biotechnological applications. Renew. Sustain. Energy Rev..

[113] Wang J., Tian Q., Cui L., Cheng J., Zhou H., Peng A., Qiu G., Shen L. (2022). Effect of extracellular proteins on Cd(II) adsorption in fungus and algae symbiotic system. J. Environ. Manag..

[114] de-Bashan L.E., Bashan Y. (2010). Immobilized microalgae for removing pollutants: Review of practical aspects. Bioresour. Technol..

[115] Pittman J.K., Dean A.P., Osundeko O. (2011). The potential of sustainable algal biofuel production using wastewater resources. Bioresour. Technol..

[116] Park J.B.K., Craggs R.J., Shilton A.N. (2011). Wastewater treatment high rate algal ponds for biofuel production. Bioresour. Technol..

[117] Craggs R., Heubeck S., Lundquist T., Benemann J. (2011). Algal biofuels from wastewater treatment high rate algal ponds. Water Sci. Technol..

[118] Kesaano M., Sims R.C. (2014). Algal biofilm based technology for wastewater treatment. Algal Res..

[119] Zeng X., Guo X., Su G., Danquah M.K., Zhang S., Lu Y., Sun Y., Lin L. (2015). Bioprocess considerations for microalgal-based wastewater treatment and biomass production. Renew. Sustain. Energy Rev..

[120] Mata Y.N., Blázquez M.L., Ballester A., González F., Muñoz J.A. (2009). Biosorption of cadmium, lead and copper with calcium alginate xerogels and immobilized *Fucus vesiculosus*. J. Hazard. Mater..

[121] Ruiz-Marin A., Mendoza-Espinosa L.G., Stephenson T. (2010). Growth and nutrient removal in free and immobilized green algae in batch and semi-continuous cultures treating real wastewater. Bioresour. Technol..

[122] Raeesossadati M.J., Ahmadzadeh H., McHenry M.P., Moheimani N.R. (2014). CO_2_ bioremediation by microalgae in photobioreactors: Impacts of biomass and CO_2_ concentrations, light, and temperature. Algal Res..

[123] Craggs R., Sutherland D., Campbell H. (2012). Hectare-scale demonstration of high rate algal ponds for enhanced wastewater treatment and biofuel production. J. Appl. Phycol..

[124] Cao S., Teng F., Lv J., Zhang Q., Wang T., Zhu C., Li X., Cai Z., Xie L., Tao Y. (2022). Performance of an immobilized microalgae-based process for wastewater treatment and biomass production: Nutrients removal, lipid induction, microalgae harvesting and dewatering. Bioresour. Technol..

[125] Changko S., Rajakumar P.D., Young R.E.B., Purton S. (2020). The phosphite oxidoreductase gene, *ptxD* as a bio-contained chloroplast marker and crop-protection tool for algal biotechnology using *Chlamydomonas*. Appl. Microbiol. Biotechnol..

[126] Tu Z., Liu L., Lin W., Xie Z., Luo J. (2018). Potential of using sodium bicarbonate as external carbon source to cultivate microalga in non-sterile condition. Bioresour. Technol..

[127] de la Broise D., Ventura M., Chauchat L., Guerreiro M., Michez T., Vinet T., Gautron N., Le Grand F., Bideau A., Goïc N.L. (2022). Scale-up to pilot of a non-axenic culture of *Thraustochytrids* using digestate from methanization as nitrogen source. Mar. Drugs.

[128] Phyu K., Zhi S., Graham D.W., Cao Y., Xu X., Liu J., Wang H., Zhang K. (2025). Impact of indigenous vs. cultivated microalgae strains on biomass accumulation, microbial community composition, and nutrient removal in algae-based dairy wastewater treatment. Bioresour. Technol..

[129] Raslavičius L., Semenov V.G., Chernova N.I., Keršys A., Kopeyka A.K. (2014). Producing transportation fuels from algae: In search of synergy. Renew. Sustain. Energy Rev..

[130] Raslavičius L., Striūgas N., Felneris M. (2018). New insights into algae factories of the future. Renew. Sustain. Energy Rev..

